# Young People’s Perceptions of Signposting in a Digital Mental Health Helpline: Mixed Methods Analysis of Cross-Sectional Data

**DOI:** 10.2196/73369

**Published:** 2026-05-19

**Authors:** Angel Yee Fong Au, Anya Jan, Shyn Wei Phua, Leslie Morrison Gutman

**Affiliations:** 1 Faculty of Brain Sciences, University College London, 1-19 Torrington Place, London, WC1E 7HB, United Kingdom, +44 2076792000

**Keywords:** internet, online, services, adolescents, youth, qualitative, survey, crisis, web-based, telephone

## Abstract

**Background:**

Mental health problems are prevalent among young people aged 16 to 24 years. With the health care system prioritizing severe cases, most young people wait months before accessing professional support. One-to-one helplines offer alternative and accessible mental health services for young people with emotional support, psychoeducation, and signposting. Signposting empowers young people to access long-term support beyond a brief helpline session. However, young people often choose not to access the signposts. Despite its importance, there is a dearth of existing research examining signposting via digital mental health helplines for young people.

**Objective:**

Using cross-sectional survey data from The Mix, a UK charity supporting young people aged 25 years or younger, this study conducted a mixed methods analysis of their multichannel (webchat, email, telephone, and web-based contact form) helpline survey between February 2020 and October 2023.

**Methods:**

The analytic sample included 296 participants who collectively received 872 signposts (approximately 872/4500, 19% of signposts provided during the survey collection period), of which 822 with complete outcome data were included in the statistical models. Multinomial logistic regressions were conducted to examine whether young people’s use and perceived usefulness of the signposts they received differed across modes of delivery and their demographic characteristics (gender, ethnicity, and age). Qualitative thematic analysis of 106 open-ended responses from 97 participants was also examined to illuminate why young people found signposting helpful and how it could be improved.

**Results:**

In the overall model, which included all predictors, webchat users identifying as White, women, and aged 16‐19 years were significantly more likely to use and find signposts helpful than to perceive them as unhelpful (odds ratios [OR] 0.28, 95% CI 0.17-0.46; *P*<.001), not intend to use them (OR 0.13, 95% CI 0.07-0.26; *P*<.001), or only plan to use them later (OR 0.29, 95% CI 0.18-0.46; *P*<.001). Thematic analysis of open-ended responses revealed that young people found the choice of signposts relevant and appreciated how signposting was integrated with emotional support. Young people also felt more hopeful after being signposted and gained both clarity and insight into the support available. However, they also noted challenges, such as feeling overwhelmed or encountering outdated signposts.

**Conclusions:**

Given the increasing reliance on digital mental health services, ensuring that signposting remains accessible, relevant, and tailored to diverse user needs is essential. By optimizing signposting strategies, helplines can empower young people to seek appropriate long-term support, ultimately improving mental health outcomes.

## Introduction

### Mental Health Support for Young People

Mental health problems explain 13% of the global burden of disease for 10- to 19-year-olds and are experienced by 14% of adolescents [1]. In the United Kingdom, 22% of young people aged 8 to 25 years experience a probable mental health problem [2]. While young people experiencing severe conditions, like psychosis or eating disorders, were seen within 2 to 4 weeks of referral [3], half of those with mild-moderate mental health problems waited 5 to 18 weeks for support [4]. Due to this delay, young people perceive care to be inaccessible and unresponsive, and therefore, can be discouraged from seeking help and often rereferred with more severe conditions [5,6]. Many times, even when support is offered after long waits, young people are more likely to refuse it or drop out [7].

Digital mental health interventions such as one-to-one helplines, peer support, and self-help resources are alternative sources of mental health support for young people [8]. Available across multiple modes of delivery (eg, text, email, phone calls, and webchat), one-to-one helplines offer brief, confidential support alongside signposting to specialist services [9,10]. Signposting encourages individuals to actively access specialist services, thus empowering them to build long-term support networks and develop healthy coping strategies beyond the helpline sessions [11]. However, many young people do not follow up on signposting [12]. Furthermore, there is a dearth of research examining young people’s experiences of signposting via digital mental health helplines, especially across various modes of delivery and in relation to young people’s demographic characteristics, information that is essential for improving the uptake and tailoring the usefulness of this service to different groups.

To help address this gap, this study examines signposting data from The Mix’s helpline, the United Kingdom’s leading digital mental health support platform for under-25s, to analyze differences in young people’s signposting experiences according to the mode of delivery and young people’s gender, ethnicity, and age. Using mixed methods, we combined quantitative findings with qualitative insights to inform recommendations to improve signposting via digital mental health helplines for young people.

### Research Background

Signposting involves a practitioner sharing resources and encouraging individuals to actively access them [12]. It requires young people to acknowledge their need for support, identify which services to access, and feel confident that those services are helpful [13]. When young people seek support, many are unaware of the services available to them [14]. This lack of awareness can act as a barrier to accessing timely and relevant support. Even when young people are aware of the services available, only a fraction actively engage and access them. To unpack how signposting may support young people beyond the immediate helpline contact, we situate this study within complementary perspectives on help-seeking and engagement. Help-seeking models (eg, Andersen’s behavioral model) conceptualize service use as shaped by (1) predisposing factors (eg, demographics and beliefs), (2) enabling factors (eg, access, knowledge of services, and perceived fit), and (3) perceived need. In this framing, signposting may function as a bridge between “in-the-moment” support and longer-term care by reducing informational barriers, increasing perceived options, and supporting readiness to act. In addition, frameworks for engagement in digital and brief interventions emphasize that uptake is more likely when support is experienced as relationally safe, autonomy-supportive, and tailored to the user’s context (eg, clear rationale, collaborative choice, and practical next steps). Together, these perspectives support interpreting signposting outcomes as a function of both service design (eg, mode of delivery and the interactional context) and user context (eg, disclosure, identity safety, and perceived accessibility of resources). For instance, Mustafa et al [12] found that while 79% (26/33) of health students knew about the available mental health services, only 27% (9/33) accessed them, highlighting the critical role practitioners play in bridging the gap, not only by sharing information, but also by motivating young people to engage with support services. Poor signposting and repeated referrals [15] can also push young people between irrelevant services, reducing their confidence in getting support.

These frameworks directly informed this study. Andersen’s model guided our focus on predisposing factors (gender, ethnicity, and age) and enabling factors (mode of delivery) as predictors of signposting outcomes, while engagement frameworks motivated our expectation that relational and interactional features of each mode would shape whether signposting felt relevant and actionable. Together, they justify a mixed methods approach—quantitative analysis to identify which user and service characteristics predict signposting outcomes, and qualitative analysis to illuminate the mechanisms that explain those patterns.

Research examining signposting has mainly focused on primary care and school settings. For example, previous qualitative studies have examined the barriers to signposting for primary care professionals in the United Kingdom, which included a lack of formal training, decreased motivation to signpost, and time constraints [16,17]. In terms of young people’s mental health, Tegethoff and colleagues [18] found that using school mental health services for signposting significantly predicted the use of external mental health services, showing the importance of schools in raising awareness of available resources. However, there is a dearth of studies examining signposting for young people’s mental health in the United Kingdom. In 1 notable exception, a qualitative study found that volunteers felt they lacked skills in person-centered signposting but benefited from the extensive signposting database [19]. This study, however, only focused on volunteers of a mental health helpline for young people delivered via email.

More research is needed to examine young people’s signposting experiences for their mental health, especially across different modes of delivery. Helplines can be delivered asynchronously (eg, email and web-based contact form) or in real-time (eg, webchat and phone). Young people prefer to have control over how they access support, and offering a multichannel helpline can encourage help-seeking [20]. Helplines via email or contact form encourage young people to explain their situation without time constraints, which is particularly effective for those experiencing social anxiety [21]. Webchats empower users to share their concerns via text messages and have live conversations with helpline agents. Similarly, phone calls allow users to express themselves synchronously with their voice. Despite these differences in user preferences, there is little or no information on whether young people’s use and their perceptions of signposts differ across modes of delivery. Such information would provide a better understanding of whether signposting is more or less well-received and used depending on how it is delivered.

There is also no current research examining whether young people’s experiences of signposting differ across their demographic characteristics. Nevertheless, past studies have found differences in young people’s use of helplines across demographic groups. Most helpline users tend to be White, women [22], and older adolescents [23], with men [24] less likely to access helplines. Due to the unavailability of culturally specific services [25], South Asian and Black Caribbean adolescents are also less likely to seek help as they worry about confidentiality [22,26] and parent surveillance or disapproval [27]. It is increasingly important to examine diversity in young people’s experiences with helplines, especially as gender and ethnic minorities [28] are not usually reported in helpline evaluations [23].

### This Study

Using secondary cross-sectional survey data from The Mix regarding their multichannel helpline (ie, webchat, email, web-based contact form, and phone), quantitative analysis was conducted to examine whether young people’s use and perceived usefulness of the signposts they received differed across modes of delivery and their demographic variables (gender, ethnicity, and age), reflecting the predisposing and enabling factors identified in help-seeking frameworks. Qualitative thematic analysis of open-ended responses was also examined to illuminate why young people found signposting helpful and how it could be improved.

## Methods

### Setting

The Mix’s one-to-one, anonymous helpline provides emotional support and information via email, webchat, telephone, and web-based contact form. It is delivered by volunteers who have received a 25-hour training. Volunteers signpost young people based on The Mix’s signposting database “The Red Book,” which compiles national and local services for diverse problem areas. Following the CHERRIES (Checklist for Reporting Results of Internet E-Surveys) checklist, we note this was a closed, postcontact web survey administered to recent users of The Mix’s multichannel helpline (webchat, email, telephone, and web contact form) between February 2020 and October 2023.

### Participants

From February 2020 to October 2023, The Mix emailed helpline users a survey link. Participation was voluntary. During this time, The Mix provided approximately 4500 signposts to users. Of the 807 participants over the age of 16 who responded to The Mix’s survey, we excluded participants with missing signposting data (n=4), those who did not complete any demographic questions (n=58), those who were not signposted (n=387), and those who were signposted but did not report any outcomes (n=63), totaling 511 excluded participants. This resulted in an analytical sample of 296 participants who collectively received 872 signposts, representing approximately 19% of the signposts provided during the survey collection period. Of these, 49 participants only partially reported signposting outcomes, creating a sample of 822 signposts included in the statistical models. The characteristics of the analytical sample are presented in Table 1.

Fisher exact test revealed significant differences in mode of delivery (*P*<.001) between the analytical sample (n=296) and those who were not signposted (n=387), with the analytical sample showing a higher proportion of phone and contact form users, but a lower proportion of email and webchat users. Compared with those who were signposted but did not report any outcomes (n=63), the analytical sample (n=296) differed significantly according to mode of delivery (*P*<.001), gender (*P*<.001), ethnicity (*P*<.001), and age (*P*=.001). Specifically, the analytical sample had a higher proportion of users aged 16‐18 and 19‐21, users identifying as White, Asian, or Mixed ethnicity, and women or other genders.

Fisher tests also revealed no demographic differences between participants who provided qualitative responses and those who did not in the signposted group (mode of delivery: *P*=.98, gender: *P*=.98, ethnicity: *P*=.99, and age: *P*=.99).

**Table 1.** Demographic characteristics of the final sample (n=296).

Characteristics	n (%)
Gender	
Women	176 (59)
Men	45 (15)
Nonbinary	3 (1)
Transgender	2 (1)
Other	9 (3)
Prefer not to say	12 (4)
Missing	49 (17)
Age (y)	
16‐19	101 (34)
20‐21	85 (29)
22‐25	55 (19)
Older than 26	9 (3)
Prefer not to say	2 (1)
Missing	44 (15)
Ethnicity	
White	189 (64)
Asian	23 (8)
Black	10 (3)
Mixed	12 (4)
Other	1 (0.3)
Prefer not to say	7 (2)
Missing	54 (18)
Mode of delivery	
Webchat	127 (43)
Contact form	96 (32)
Email	54 (18)
Phone	19 (6)

### Procedure

From February 2020 to October 2023, all helpline users aged 16 to older than 25 years who contacted the helpline were asked to complete their user survey via email. After clicking the survey link, participants read an information sheet explaining the research aims. Informed consent was then obtained online. Only survey responses from those aged 16 or above were included in this study in line with ethical guidelines for informed consent. Upon providing informed consent, participants completed the online survey on SmartSurvey (Appendix S1 in Multimedia Appendix 1).

This study analyzed only the demographic data and questions related to signposting. The survey platform, number of pages or items, use of randomization or skip or branching, presence of completeness checks, and whether answers could be reviewed or changed before submission were not specified.

### Measures

#### Demographic Questions

Modes of delivery included webchat, contact form, email, and phone. Gender options included women, men, nonbinary, transgender, other, and prefer not to say. Consistent with the ethnic groups in the UK Census [29], ethnicity options included 19 ethnic groups. Since at least 10 observations per predictor group are required for multinomial logistic regressions to yield reliable findings [30], ethnicity was collapsed into 6 broad groups—White, Asian, Black, Mixed, Other, and prefer not to say. Age was collapsed into 16‐19 years (further education and transition into adulthood), 20‐21 years (higher education and graduation), 22‐25 years (postgraduate education and employment), older than 26 years (caregivers or professionals), and prefer not to say.

#### Signposting Outcomes

For the young person’s use and their perception of the usefulness of each signpost, as well as their overall signposting experience, the survey included multiple-choice and open-ended questions, outlined in Table 2. In line with CHERRIES, items comprised fixed-response options plus open-ended questions on experience and improvement.

**Table 2.** Survey questions on signposting outcomes.

Question types	Questions
Multiple-choice question on the outcome for each signpost	Did you find the below organizations or resources recommended to you useful? Yes (Used it and found it useful) No (Used it and found it not useful) I haven’t used it but I’m planning to do so I haven’t used it and NOT planning to do so Not relevant
Open-ended questions for each participant	Please tell us how the service helped you. How can we improve? Please let us know if you have any other comments or feedback.

### Data Analysis

#### Quantitative Analysis

As a closed survey, the view rate is not applicable. Participation and completion rates were not reported. Analyses used the analytic samples described below. Addressing each signpost as an independent item, item-wise analysis was carried out to explore whether demographic variables (categorical) predicted young people’s use or perceived usefulness of the signposts (categorical). Using the *mblogit* function from the *mclogit* package in R (R Core Team) [31], multinomial logistic regressions were conducted with only the fixed effects of mode of delivery, gender, ethnicity, and age on signposting outcomes, alongside an overall model with fixed effects of all variables. “Yes” (useful) was the reference category. Although this outcome was a repeated measure depending on the number of signposts for each participant, the model could not converge after adding the random intercept of participants. Nonconvergence may reflect the complexity of the multinomial outcome and limited sample sizes in certain categories. As a result, we reported fixed-effects models treating each signpost outcome as an item-level observation. This decision prioritized model stability and interpretability given sparse cells, but may underestimate uncertainty because outcomes are clustered within participants; findings should therefore be interpreted as exploratory associations.

#### Qualitative Analysis

Inductive, critical realist thematic analysis [32] was conducted. Responses relevant to signposting from the open-ended questions were first shortlisted by filtering responses from participants who were signposted, then searching for keywords (eg, “link,” “resource,” “signpost,” “organisation,” and “website”), and reading the remaining responses to include relevant ones without the keywords (eg, *“*I decided to book an appointment with my GP to get help*”*).

From these shortlisted responses, 18 initial codes were developed and later organized into 4 main themes using NVivo 14 (Lumivero) [33]. Themes emerged from responses reflecting similar signposting experiences or impacts, with smaller subthemes representing recurring concepts. A codebook with 4 themes and 12 subthemes was eventually created (Appendix S2 in Multimedia Appendix 1).

With varying length and depth of responses, some were coded to more than 1 subtheme. For intercoder reliability, 25 responses were independently coded. The percentage of agreement (ie, proportion of items assigned to the same codes by both researchers) was 76%, indicating substantial agreement between the coders even when responses were mapped to multiple codes [34].

To compare differences in the qualitative feedback across demographic groups, simple sentiment analysis was also conducted by contextually reading each response and annotating it as expressing either positive or negative sentiment. Positive responses were identified as those expressing satisfaction, perceived benefits, or motivation to engage with signposts (eg, “I found a really helpful support group”). Negative responses were categorized as those highlighting barriers, dissatisfaction, or challenges (eg, “the signposted resources didn’t meet my needs”). Responses were contextually read and annotated accordingly. Coding of sentiment was conducted manually, with annotations guided by an agreed-upon framework to ensure consistency.

### Ethical Considerations

Ethical approval was granted by the University College London Research Ethics Committee (ethics ID: 16583/003). Participants first viewed an online information sheet and then provided informed consent before answering any items. To protect participant privacy, all survey data were fully anonymized, and no personally identifiable information was collected or retained. As compensation for their time, participants were entered into a prize draw with the chance to win a £50 (US $66.64) Amazon voucher.

## Results

### Overview

Due to varied levels of data missingness for demographic variables, observations with missing data were removed before analyzing each predictor (ie, participants with missing data for ethnicity were only excluded from analyses on ethnicity).

### Quantitative Analysis: Signposting Outcomes

As illustrated in the Appendix S3 in Multimedia Appendix 1, individual models were first conducted for the mode of delivery (n=822), gender (n=684), ethnicity (n=669), or age (n=697) before running an overall model with all predictors included (n=669). The individual models demonstrated patterns broadly consistent with the overall model, with some notable differences. The mode of delivery model highlighted that webchat was the most effective channel for signposting outcomes, with other platforms (contact form, email, and phone) associated with a lower likelihood of participants using and finding signposts helpful. The gender model showed that participants identifying as other genders or those who preferred not to disclose their gender were less likely to find signposts helpful, with a greater likelihood of reporting irrelevance or a delayed intention to use them. The ethnicity model revealed that individuals who preferred not to disclose their ethnicity were significantly more likely to perceive signposts as unhelpful or irrelevant, a trend more pronounced than in the overall model. The age model showed that older participants (20‐25 y) were less likely to find signposts helpful, with these effects being slightly more potent in the individual model than when controlling for all predictors in the overall model. In the overall model, which included all predictors, webchat users identifying as White, women, and aged 16‐19 years were significantly more likely to use and find signposts helpful than to perceive them as unhelpful (odds ratio [OR] 0.28, 95% CI 0.17-0.46, *P*<.001), not intend to use them (OR 0.13, 95% CI 0.07-0.26, *P*<.001), or only plan to use them later (OR 0.29, 95% CI 0.18-0.46, *P*<.001). These results demonstrate the importance of both the delivery mode and participant demographics in predicting the perceived effectiveness of signposting.

Of 669 signposts in the overall model, 360 (54%) were given via webchat, 480 (72%) were shared with participants who identified as women, 521 (78%) with White participants, and 350 (52%) with 16- to 19-year-olds. Thus, webchat, women, White, and 16- to 19-year-olds were the reference categories for the multinomial logistic regressions.

A multinomial logistic regression examined whether mode of delivery, gender, ethnicity, or age predicted signposting outcomes. A significant chi-square difference test (*F*_64_=181.27, *P*<.001) indicated that our model significantly improved upon the null. The model explained 43% of the variance (Nagelkerke *R*^2^) with a low Akaike Information Criterion (AIC=1934) and Bayesian Information Criterion (BIC=2240).

Shown in Table 3 as the intercept, webchat users identifying as White, women, and 16- to 19-year-olds were significantly more likely to use the signposts and find them useful than perceiving them as unhelpful (OR 0.28, 95% CI 0.17-0.46; *P*<.001), not intending to use them (OR 0.13, 95% CI 0.07-0.26; *P*<.001), or only planning to use them (OR 0.29, 95% CI 0.18-0.46; *P*<.001). This reference group, therefore, represented the most favorable signposting outcomes in the model.

Compared with webchat users, users accessing other platforms (contact form, email, and phone) were less likely to use and find the signposts helpful. Contact form users were more likely not to plan to use the signposts (OR 4.98, 95% CI 2.29-10.83; *P*<.001), only plan to use them later (OR 3.95, 95% CI 1.98-7.89; *P*<.001), or find them irrelevant (OR 2.90, 95% CI 1.60-5.25; *P*<.001). Email users were more likely to only plan to use the signposts (OR 2.77, 95% CI 1.50-5.13; *P*=.001). Phone users were even more likely to have used the signposts but find them unhelpful (OR 4.17, 95% CI 1.58-11.00; *P*=.004).

Compared with women, participants identifying as other genders or preferring not to say their gender were less likely to find signposting useful. Those identifying as other genders were more likely to plan to use signposts later (OR 8.46, 95% CI 2.13-33.66; *P*=.002) or find them irrelevant (OR 4.49, 95% CI 1.22-16.59; *P*=.02). Participants who preferred not to disclose their gender were more likely to have no intention to use the signposts (OR 3.82, 95% CI 1.19-12.26; *P*=.03).

Compared with White participants, those who preferred not to share their ethnicity were more likely to find the signposts unhelpful (OR 45.97, 95% CI 3.56-594.15; *P*=.003) or irrelevant (OR 16.95, 95% CI 1.50-191.26; *P*=.02). The wide CIs reflect the small sample size for this group and should be interpreted with caution.

Compared with 16- to 19-year-olds, 20- to 25-year-olds were less likely to find signposts helpful. 20- to 21-year-olds were more likely to only plan to use the signposts (OR 3.13, 95% CI 1.64-5.97; *P*=.001) or perceive them as irrelevant (OR 1.80, 95% CI 1.01-3.20; *P*=.045), while 22- to 25-year-olds were more likely to not plan to use them (OR 2.92, 95% CI 1.30-6.53; *P*=.009) or find them irrelevant (OR 1.80, 95% CI 1.05-3.08; *P*=.03). This suggests engagement with signposting decreases progressively across 20- to 25-year-olds.

**Table 3.** Multinomial logistic regression on signposting outcomes and all predictors (n=669)^a^.

Predictors	Useful: no		Useful: not used and no intent		Useful: not used but has intent		Useful: not relevant	
	Odds ratios (95% CI)	*P* value	Odds ratios (95% CI)	*P* value	Odds ratios (95% CI)	*P* value	Odds ratios (95% CI)	*P* value
Intercept	0.28 (0.17‐0.46)	<.001	0.13 (0.07‐0.26)	<.001	0.29 (0.18‐0.46)	<.001	0.77 (0.54‐1.10)	.15
Mode of delivery								
Contact form versus webchat	1.82 (0.78‐4.26)	.17	4.98 (2.29‐10.83)	<.001	3.95 (1.98‐7.89)	<.001	2.90 (1.60‐5.25)	<.001
Email versus webchat	1.69 (0.83‐3.44)	.15	0.14 (0.02‐1.05)	.06	2.77 (1.50‐5.13)	.001	1.24 (0.72‐2.15)	.44
Phone versus webchat	4.17 (1.58‐11.00)	.004	0.00 (0.00-Inf)	>.99	0.83 (0.24‐2.93)	.78	0.99 (0.38‐2.60)	>.99
Gender								
Men versus women	0.63 (0.29‐1.40)	.26	1.87 (0.80‐4.36)	.15	1.35 (0.71‐2.58)	.37	1.07 (0.61‐1.88)	.81
Nonbinary or transgender versus women	0.00 (0.00-Inf)	>.99	0.00 (0.00-Inf)	>.99	0.34 (0.06‐1.90)	.22	0.40 (0.11‐1.43)	.16
Other versus women	0.00 (0.00-Inf)	>.99	0.00 (0.00-Inf)	>.99	8.46 (2.13‐33.66)	.002	4.49 (1.22‐16.59)	.02
Prefer not to say versus women	0.00 (0.00-Inf)	>.99	3.82 (1.19‐12.26)	.03	0.85 (0.26‐2.72)	.78	0.86 (0.31‐2.40)	.77
Ethnicity								
Asian versus White	2.40 (0.92‐6.29)	.08	0.88 (0.25‐3.06)	.84	1.37 (0.54‐3.47)	.50	2.05 (0.95‐4.42)	.07
Black versus White	1.56 (0.36‐6.75)	.55	0.82 (0.09‐7.77)	.86	2.00 (0.59‐6.72)	.26	1.66 (0.56‐4.91)	.36
Mixed versus White	1.72 (0.47‐6.31)	.42	2.07 (0.47‐9.10)	.34	0.69 (0.18‐2.61)	.59	2.06 (0.81‐5.25)	.13
Other versus White	1.89 (0.00-Inf)	>.99	3.79 (0.00-Inf)	>.99	1.12 (0.00-Inf)	>.99	88660893.75 (0.00-Inf)	>.99
Prefer not to say versus White	45.97 (3.56‐594.15)	.003	0.00 (0.00-Inf)	>.99	3.17 (0.14‐70.30)	.47	16.95 (1.50‐191.26)	.02
Age (y)								
20‐21 versus 16‐19	1.89 (0.89‐4.00)	.10	1.96 (0.78‐4.93)	.15	3.13 (1.64‐5.97)	.001	1.80 (1.01‐3.20)	.045
22‐25 versus 16‐19	1.73 (0.86‐3.48)	.12	2.92 (1.30‐6.53)	.009	1.46 (0.75‐2.86)	.26	1.80 (1.05‐3.08)	.03
Older than 26 versus 16‐19	0.82 (0.15‐4.48)	.82	0.80 (0.09‐7.42)	.84	1.08 (0.28‐4.15)	.91	1.00 (0.31‐3.24)	>.99
Prefer not to say versus 16‐19	0.00 (0.00-Inf)	>.99	0.00 (0.00-Inf)	>.99	0.00 (0.00-Inf)	>.99	0.08 (0.01‐1.14)	.06

a Note. Nagelkerke *R*2=0.430. Extremely large odds ratios and wide CIs (eg, 0.00 to infinity) may result from zero or small sample sizes for comparisons. Nonbinary (n=8) and transgender (n=6) were collapsed due to small sample sizes. Other ethnicities (n=3) and those who preferred not to say their age (n=4) were not collapsed due to the distinct theoretical implications, but relevant findings should be interpreted with caution.

### Qualitative Analysis

Of 358 participants who were signposted to at least 1 resource, 257 participants submitted 441 responses across 3 open-ended questions. Among these, 106 responses from 97 participants relevant to signposting (eg, “Sent me some links to places to help”) were analyzed. Among 106 responses, 76 (72%) indicated positive sentiment, while 30 (28%) were negative. Furthermore, 4 main themes were identified: “choice of signposts,” “signposting experience,” “responses to signposting,” and “effectiveness of signposting.” Table 4 presents the definitions, examples, and number of responses across themes. Note that each response could be coded to multiple subthemes.

**Table 4.** Definitions, examples, and number of responses across themes and subthemes.

Theme and subtheme	Response type and examples
Theme 1: choice of signposts	Comments specific to the signposts that influence young people’s perception of whether signposting was effective
Relevance (n=8)	Whether the signposts were relevant to the young people’s specific concerns “provided some websites and organizations which would help me with my issue” “Recommend me to an organization that can help me with my bank account issue”
Personalized (n=14)	Whether the signposts were specific to the young people’s demographics (eg, geographical location, age, and support needs) “clear and useful about what would be available to someone my age” “Make note of the personal details I sent to you and tailor the help offered with those details”
Expectation (n=5)	Whether the signposts met young people’s other expectations (eg, number, features, and specific requests) “an app would be good with some resources”
Theme 2: signposting experience	Comments specific to how signposting was completed by helpline volunteers
Integration of emotional support with signposting (n=30)	How signposting was integrated with offering emotional support by the volunteers “They understood what I was going through and gave me organizations to look at” “…feel a bit demoralizing…better balance between providing some level of support and recommending services”
Elaboration on signposts (n=4)	Whether sufficient details of signposts were provided to young people “…gave detailed explanations of when and how to use them” “…explain more about what the websites are”
Preference for additional support (n=5)	Preference for receiving additional support such as direct referrals “Employ some experts in the field of sexual assault…” “contact the other agency with you”
Theme 3: responses to signposting	Immediate responses to signposting during or shortly after the chat
Hopefulness and direction (n=14)	Signposting inspiring hope and a sense of direction “…gave me hope that things will improve” “…pointing me in the right direction of where to go”
Rejection and overwhelm (n=7)	Signposting leading to feelings of rejection or overwhelm “…felt like most comments…was a lead-in to recommend a specific service” “I’m struggling to get through the list”
Theme 4: effectiveness of signposting	Effects of signposting on young people’s well-being after the chat has ended
Insights into resources (n=53)	Enhanced knowledge of resources for young people “…made me aware of some local resources that I didn’t know was available”
Empowerment (n=13)	Enabling young people to learn more about their situation and seek support “…managed to get some plan figured out for myself and scheduled appointments” “feel a lot more confident in myself”
Access to signposts (n=10)	Ease for young people to access the signposts “helplines provided to me were either no longer available or outdated” “helped me to access the resources I needed”
Impacts on well-being (n=5)	Long-term mental health outcomes from signposting “…helped not to kill myself” “received counseling to get past…a time where I felt lost”

### Theme 1: Choice of Signposts

#### Relevance

Participants appreciated the relevance of signposts to their concerns, while some critiqued their relevance. Participants valued the volunteers’ effort in selecting the most pertinent signposts: “They also took the time to make sure they directed me to the most relevant resources.” However, several participants highlighted that volunteers sometimes misunderstood their needs and shared less suitable signposts. For instance, “The websites that were recommended for me […] were to give me more information on my situation, rather than what I could do next to improve.”

#### Personalized

Some participants recognized how signposts were tailored to their age, location, or support needs, whereas others found them too general. Participants liked it when volunteers inquired about their needs before signposting (“before recommending other services, I was asked how I’d like to contact people”). However, some felt that the volunteers failed to personalize the signposts despite their efforts to communicate their needs (“Make note of the personal details I sent to you and tailor the help offered”).

#### Expectation

Responses mentioned specific requests, such as being signposted to apps (*“*an app would be good with some resources”).

### Theme 2: Signposting Experience

#### Integration of Emotional Support and Signposting

Participants felt heard alongside being signposted (“felt really listened to and actually heard”), while others expressed dissatisfaction with the signposting experience. Participants appreciated being “welcomed with compassion and understanding in very quick time frames” and the nonjudgmental space to share (“gave me the space to get things off my chest”). Yet, some responses suggested that the emotional support felt scripted (“might have been [an] automatic response”) and “a lead-in to recommend a specific service.” One participant noted the imbalance between emotional support and signposting: “Try not to solely rely on signposting loads of organisations, as it can feel a bit demoralising to just be moved onto someone else.”

#### Elaboration on Signposts

Some responses valued detailed explanations of signposts, while others anticipated further information. Participants appreciated how the volunteers “gave detailed explanations of when and how to use [the signposts],” while others wanted volunteers to “[explain] what each thing could do, such as explaining what counselling is and what it would benefit.”

#### Preference for Additional Support

Responses indicated a preference for additional support, such as in-house specialists (*“*Employ some experts in the field of sexual assault, rape”), more partnerships (“working with more [organizations]”), or direct referrals. One participant suggested that direct referrals may be more effective than signposting because young people may not be well-informed enough to access support: “Make the referral for [the person] because they may be confused and not do it, putting themselves at more of a risk.”

### Theme 3: Responses to Signposting

#### Hopefulness and Direction

Participants illustrated that signposting inspired hope and a sense of direction. Before using the helpline, participants were desperate for support (“I was really desperate for some help and you gave it to me”*).* Signposting then led them to feel more hopeful about the future (“given me some hope that improvement to my situation is possible”) and seek help (“I hope at least one or more [of the signposts] will be helpful”). Additionally, some acknowledged how signposting guided them to appropriate services *(*“point me in the right directions to organizations and resources”), and they “don’t feel stuck again.”

#### Rejection and Overwhelm

Participants conveyed feelings of rejection or overwhelm. Several participants felt dismissed to other services and regretted seeking help: “I was just being referred to other services, so I am quite unhappy that my reaching out didn’t pay off as I feel like I’m not gonna get helped.” Another emphasized that the excessive number of signposts demotivated them from seeking support: “I was overwhelmed by the amount of other places to look for help that I’m struggling to get through the list.”

### Theme 4: Effectiveness of Signposting

Effective signposting empowers young people to learn about, trust, and take action to access support [13]. Subthemes were developed to reflect these aspects.

#### Insights Into Resources

Many participants discovered new local resources (“[…] made me aware of some local resources that I didn’t know was available”) or found suitable resources (“helped me to find the resources to get the help I need”).

#### Empowerment

Participants demonstrated how signposting empowered them to express themselves and seek help. They found their situations more manageable (“Made me feel in control of my situation”) and became confident to open up (“I had the courage to speak up to someone about how I felt”). Some took immediate action to access support: “With only contacting the helpline yesterday I have already managed to get some plan figured out for myself and scheduled appointments.”

#### Access to Signposts

Participants critiqued that signposts were inaccessible, although 3 accessed the suitable signposts (“helped me to access the resources I needed”). Furthermore, 6 participants were unable to use The Mix’s counseling services due to unavailability: “I did want telephone counselling but the referral pathway was closed due to no space.” One highlighted other signposts were outdated: “helplines provided to me were either no longer available or outdated.”

#### Impacts on Well-Being

Participants shared that the signposts improved their well-being (“I still use the app that they suggested to me, and so far it’s been helping”). Signposting helped participants navigate difficult times (“received counseling to get past […] a time where I felt lost”) and was even lifesaving (“It helped found myself and the right people to go. It helped not to kill myself”).

### Connections With Quantitative Findings

A breakdown of the participants’ demographics and the sentiment of their responses is presented in Table 5. Regarding the mode of delivery, email (n=12, 63%), telephone (n=4, 67%), and contact form (n=34, 68%) showed similar levels of positive sentiment, while webchat had the highest proportion of positive sentiment (n=26, 84%). Email helpline users mentioned that signposts were given too quickly *(*“Through email, take time to work through each issue, rather than quickly offering other organisations”) while others reported otherwise *(*“With the email I can take my time and follow each link”). Contact form users had mixed sentiments, with some appreciating its asynchronous delivery (“helpful in visualising and stepping away from my issue”) and others dissatisfied with feeling pushed away *(*“[…] actually help me rather than referring”). A few phone helpline users commented on the relevance of signposts (“Provide more relevant support services”).

**Table 5.** Demographics characteristics and sentiments of the open-ended responses (n=106).

Characteristics	Positive, n/N (%)	Negative, n/N (%)
Gender		
Women	38/55 (69)	17/55 (31)
Men	11/16 (69)	5/16 (31)
Nonbinary	2/2 (100)	0/2 (0)
Transgender	1/1 (100)	0/1 (0)
Other	3/3 (100)	0/3 (0)
Prefer not to say	2/4 (50)	2/4 (50)
Missing	19/25 (76)	6/25 (24)
Age (y)		
16‐19	30/45 (67)	15/45 (33)
20‐21	9/11 (82)	2/11 (18)
22‐25	16/23 (70)	7/23 (30)
Older than 26	3/3 (100)	0/3 (0)
Prefer not to say	1/1 (100)	0/1 (0)
Missing	17/23 (74)	6/23 (26)
Ethnicity		
White	47/66 (66)	19/66 (66)
Asian	6/9 (67)	3/9 (33)
Black	0/2 (0)	2/2 (100)
Mixed	1/1 (100)	0/1 (0)
Other	0/0 (0)	0/0 (0)
Prefer not to say	2/2 (100)	0/2 (0)
Missing	20/26 (77)	6/26 (23)
Mode of delivery		
Contact form	34/50 (68)	16/50 (32)
Email	12/19 (63)	7/19 (37)
Phone	4/6 (67)	2/6 (33)
Webchat	26/31 (84)	5/31 (16)

Regarding gender and ethnicity, the same proportion of participants who identified as women (38/55, 69%) and men (11/16, 69%) reported positive sentiments, while 100% (6/6) of those identifying as nonbinary (n=2), transgender (n=1), or other genders (n=3) expressed positive sentiments. Of those who preferred not to say their gender (n=4), 2 (50%) expressed positive sentiment. White (n=47, 71%) and Asian (n=6, 67%) participants showed similar levels of positive sentiments, but there were 100% positive responses among those identifying as mixed ethnicity (n=1) or who preferred not to say (n=2). Black participants exclusively left negative responses (n=2, 100%), with one hoping for support from in-house experts in sexual assault and another commenting on access to The Mix’s internal counseling services.

In terms of age, responses from 16- to 19-year-olds (n=30, 67%) and 22- to 25-year-olds (n=16, 70%) had a similar percentage of positive sentiments, compared with much higher proportions of 20- to 21-year-olds (n=9, 82%), older than 26-year-olds (n=3, 100%), and those who preferred not to say (n=1, 100%). Several responses are directly related to age. For example, a 22- to 25-year-old preferred more age-tailored signposts *(*“more information about […] different services that are available to young people”), while an older than 26-year-old parent appreciated the helpline (“My daughter is really struggling. Just knowing I contacted someone helped her”).

## Discussion

### Overview

Our study revealed that participants generally perceived signposts as helpful or were at least motivated to access them after using the helpline, illustrating how signposting may empower young people to seek relevant support. Signposting outcomes varied across modes of delivery, with webchat showing the strongest association with positive outcomes, the contact form yielding mixed results, and email or phone showing weaker associations. Similar outcomes were observed across gender, ethnicity, and age, although certain minority groups (eg, those preferring not to say their gender or ethnicity, 20- to 25-year-olds) were more likely to ignore or find signposts irrelevant.

Qualitative thematic analysis indicated that participants generally found the signposts relevant and personalized. They valued volunteers’ efforts in listening actively and explaining the signposts, which appeared to inspire hope and motivate participants to access support. However, some felt bombarded with too many signposts, leading to feelings of rejection or overwhelm, potentially reducing their intention to use signposts. Those discouraged from accessing signposts often faced barriers such as outdated information, explaining why some participants may have used signposts but found them unhelpful.

### Mode of Delivery

The mode of delivery was significantly associated with participants’ perceptions of usefulness and their use of signposts, underlining their benefits and disadvantages for young people. Across the 4 modes, webchat was associated with the strongest positive outcomes, with participants perceiving signposts as relevant, trustworthy, and helpful. Real-time interactions with volunteers via webchats may facilitate collaborative therapeutic relationships by allowing young people to control the pace of disclosure, without the social pressure to respond instantly in phone calls. This may empower young people to share emotionally challenging experiences [35], such as suicidality, thus helping volunteers to identify the most suitable signposts. Although helpline contact is brief, relational processes, such as emotional containment, trust-building, and perceived autonomy, are central to whether signposting feels safe and usable. Our qualitative themes suggest that when young people feel heard and validated, signposting is more likely to be experienced as supportive rather than overwhelming. These relational processes are particularly important for young people at higher risk, such as those disclosing suicidality or experiencing a mental health crisis. Volunteers should be trained to prioritize risk assessment and immediate safety planning, ensuring that signposting or referrals to external services complement rather than replace immediate support.

The contact form showed weaker associations, with signposts often perceived as irrelevant. The contact form requires users to explain their situation and receive one-off support asynchronously. For this reason, the degree of personalization in signposts may be limited by the amount of information the users share [19], causing some to perceive the signposts as less tailored.

Similarly, email support was associated with less positive outcomes for young people, possibly due to its asynchronous nature, with participants not using the signposts but intending to. Limited information may affect volunteers’ ability to select appropriate signposts, lowering their confidence to provide appropriate support via email [19]. Volunteers’ reduced emotional connection to users who contact via email [19] may further encourage volunteers to focus on signposting rather than taking more time to understand their issues, which our findings suggest may lead to some user dissatisfaction with their signposts and email support, more generally.

Despite positive reception in the existing research [36], the phone helpline in our study mainly offered 1 signpost per user, which was often perceived as unhelpful. Potential power imbalances between volunteers and users may explain this finding. Telephone helplines enhance the authority of volunteers [35], possibly reducing young people’s sense of control in the conversation, and they could agree to signposts that they may not find beneficial [20].

### Integrating Findings Across Methods

 Our qualitative themes help interpret why webchat was associated with more favorable signposting outcomes. Webchat had the highest proportion of positive sentiment (26/31, 84%), compared with 63%‐68% across other modes. Young people most often described signposting as helpful when it was embedded within an emotionally validating interaction (refer to Integration of Emotional Support With Signposting section under Theme 2) and when resources felt personally relevant rather than generic (refer to Personalized section under Theme 1). Webchat may support this because synchronous text-based conversation allows users to disclose at their own pace while also enabling volunteers to check understanding, collaboratively narrow options, and immediately clarify barriers to follow-through (eg, eligibility, waiting lists, and how to access), conditions that facilitate both personalization and emotional integration. Where these conditions were absent, young people described feelings of rejection or overwhelm (refer to Rejection and Overwhelm section under Theme 3), or found signposts insufficiently tailored to their demographics or circumstances (refer to Relevance section under Theme 1). This relational “fit” may be harder to achieve in asynchronous modes (email or contact form) where reduced back-and-forth can limit tailoring, and in phone-based interactions where perceived authority dynamics may reduce collaborative choice.

### Gender

Signposting was generally associated with positive outcomes across genders, including women, men, nonbinary, and transgender young people. Those identifying as “other” tended to either plan to use the signposts or find them irrelevant, whereas those preferring not to disclose their gender were more likely not to use the signposts. Gender minorities, who may be hesitant to seek help from general services, could find signposting valuable for discovering lesbian, gay, bisexual, transgender, queer or questioning, and others (LGBTQ+) informed services [37], boosting their confidence in accessing support [13]. Nonetheless, as existing LGBTQ+ services may not meet the needs of all, some may find their signposts irrelevant. Participants who preferred not to share their gender might have received less relevant signposts, as volunteers had insufficient information to identify suitable services. Preferences not to disclose gender may reflect broader concerns about confidentiality and stigma, as well as uncertainty about whether the service is gender-affirming, rather than simply a lack of relevance of the signposts. While the open-ended responses seem to indicate that young people who identified as nonbinary, transgender, or other were extremely positive about The Mix’s signposting, the sample sizes for gender minorities were notably small (6 to 25 signposts, 6 to 17 participants), requiring further research to validate our findings. Nondisclosure of gender or ethnicity may reflect safety and trust considerations. Qualitative comments suggest that perceived cultural fit and confidentiality concerns shape willingness to engage with signposts.

### Ethnicity

Signposting was generally associated with positive perceptions across ethnicities, although participants preferring not to say their ethnicity were more likely to find signposts unhelpful and irrelevant. For those who preferred not to disclose their ethnicity and found signposts unhelpful, volunteers may have lacked sufficient personalized information to customize their signposts. As with gender, nondisclosure of ethnicity may reflect that building trust with marginalized groups can take longer due to broader issues related to privacy, stigma, and cultural norms around help-seeking, alongside previous experiences of discrimination when seeking support, causing some to withhold their details [22,26]. In our qualitative analysis, Black participants critiqued the signposts; one mentioned limited access to counseling, and the other preferred receiving sexual assault support within The Mix rather than being signposted elsewhere, demonstrating that access to support remains a major barrier to help-seeking. Overall, the results suggest a need to regularly review and update the signposting database to provide more culturally specific and tailored services. Nonetheless, sample sizes for other ethnicities are remarkably small (3 signposts, 14 participants), so these findings should be interpreted cautiously. More broadly, nondisclosure can be understood as an equity signal. When services feel uncertain in cultural safety, young people may reduce identifiable data-sharing. Service design should therefore treat demographic “prefer not to say” responses not as missingness alone, but as potentially meaningful indicators of trust, perceived stigma, and safety.

### Age

Signposting was generally helpful across age groups, yet 20- to 25-year-olds were more likely to find signposts irrelevant. 22- to 25-year-olds were more inclined not to use the signposts, while 20- to 21-year-olds would plan to use them. Our qualitative findings suggest that these results indicate that more age-tailored support is needed. For example, 20- to 21-year-olds could be signposted to student support and external services [38], while 22- to 25-year-olds may require more services focused on the transition from school to work, relationship advice, and parenting. Notably, advice-seeking users older than 25 years were often caregivers or professionals seeking advice for young people.

### Limitations

There are several limitations to this study. First, since the participants were recruited by emailing helpline users the survey link, self-selection bias may have influenced our results [39]. Those having strong opinions about the helpline may have been more likely to share their feedback, hence skewing the data. Additionally, listwise deletion based on missing demographic data could have retained participants who were more comfortable or engaged with the service. Future studies should consider using stratified sampling to ensure that demographic groups of interest are sampled equally. Second, as we used survey data from a general UK-based helpline for young people, our analytic sample, 64% White (n=189) and 74% (n=221) identifying as men or women, was representative of the user demographic at The Mix, which is 82% (24,158/29,389) White and 97% (34,053/35,215) men or women. Due to the small percentages of ethnic and gender minorities, we collapsed categories for the quantitative analyses. Inflated ORs were still found, as not all minority groups have sufficient observations for each signposting outcome. Future research should focus on gender, ethnicity, and sexuality to enrich the existing findings, conducting focus groups or interviews for more in-depth qualitative analysis.

Furthermore, the outcomes measured in the survey may not be easily compared with outcomes used by other helplines, such as quantitative, self-report measures (eg, study by Law et al [40]). As both our study and other helplines tend to rely on retrospective self-report, recall bias may have affected the accuracy of participants’ responses. To understand the different stages of signposting [13], additional research could split the question on signposting into 4 subquestions asking participants whether they found the signposts relevant, whether they planned to use them, whether they accessed the signposts, and whether the signposts were helpful if they used them.

### Recommendations

These findings highlight the critical role of signposting in supporting young people’s mental health, particularly through webchat, which emerged as the most effective mode of delivery. Given the increasing reliance on digital mental health services, ensuring that signposting remains accessible, relevant, and tailored to diverse user needs is essential. By optimizing signposting strategies, helplines can empower young people to seek appropriate long-term support, ultimately improving mental health outcomes.

Given the mixed findings of other communication channels (eg, email, contact form, and phone), enhancing volunteer training in these areas is recommended. Training should focus on balancing emotional support and signposting in asynchronous communication, as well as effectively navigating suitable signposts during phone-based interactions [13]. For webchat, volunteers should be trained to use brief collaborative checks (eg, confirming priorities, offering 2‐3 best-fit options, and checking practical barriers such as eligibility, cost, and waitlists) so that signposting feels tailored and actionable rather than overwhelming. For email and contact-form interactions, templates that include a short, personalized rationale for each resource and clear “next-step” instructions (eg, who to contact and what to say) may improve perceived relevance and follow-through.

Additionally, periodic reviews of the signposting database are essential to ensure signposts are up to date and comprehensive. Detailed descriptions of available signposts (eg, referral requirements, waitlist lengths, and access pathways) can enhance the user experience and improve the perceived relevance of signposts [16]. Where feasible, services could also provide an “actionable signpost pack,” particularly suited to asynchronous modes, such as email and contact form (eg, 1‐3 links plus a brief explanation of why each option fits, what to expect next, and how to access), to reduce cognitive load and increase the likelihood of uptake. Expanding the database to include additional services tailored to ethnic minorities and older youth (20- to 25-year-olds) is also critical, as these groups were identified as having unique needs that may not be adequately addressed by existing services [14]. To further support marginalized groups, services could train volunteers to introduce optional demographic questions as a tool for personalizing support rather than an administrative requirement, and to incorporate explicit affirmations of cultural safety and inclusivity at the start of interactions.

Finally, integrating feedback mechanisms into the signposting process can ensure continuous improvement and adaptability to the evolving needs of young people. As part of routine quality improvement, services could monitor signposting outcomes by mode of delivery and patterns of nondisclosure, treating increases in “not relevant” responses or high “prefer not to say” rates as potential signs to review training, cultural safety, and the signposting database. For webchat, real-time postsession feedback prompts may be the most feasible. For asynchronous modes, follow-up survey links could be embedded in email responses. Periodic evaluations of these mechanisms will allow helplines to refine their signposting strategies, ensuring they remain effective and user-centered. Strengthening these approaches will not only enhance engagement but also reinforce the role of helplines as a vital bridge between immediate support and long-term health care.

## Supplementary material

10.2196/73369 Multimedia Appendix 1 Survey, codebook, and individual models.

10.2196/73369 Checklist 1 CHERRIES checklist.

## Abbreviations

CHERRIES: Checklist for Reporting Results of Internet E-Surveys
LGBTQ+: lesbian, gay, bisexual, transgender, queer or questioning, and others
OR: odds ratio
