# Supplementary material for: Young People’s Perceptions of Signposting in a Digital Mental Health Helpline: Mixed Methods Analysis of Cross-Sectional Data
Source: JMIR Hum Factors. 2026 May 19;13:e73369. doi: 10.2196/73369 (PMC13186521; doi:10.2196/73369)
Supplement: Multimedia Appendix 1 [file humanfactors-v13-e73369-s001.docx]

## Appendix S1

Helpline Feedback Survey from The Mix

1. Introduction

Thank you for contacting The Mix helpline in the last few days. We are always thinking about improving our services. Please complete this short survey about your experience with The Mix helpline to help us understand how we can improve.

Take this short survey and you could win a £50 Amazon voucher.

Everything you say is confidential and held in line with our privacy policy, accessible here: https://goo.gl/7wrsDF

By selecting the "Start the survey" option below you agree to participate in the survey by The Mix to help us improve. You agree that you are 13 years old or older. *

Start the survey

I am 12 years old or younger

Total number of Signposts

Signpost 1 Number

Signpost 1 Name

Signpost 2 Number

Signpost 2 Name

Signpost 3 Number

Signpost 3 Name

2. Thank you

Thank you for your interest. We are sorry but due to GDPR regulations and your age we are unable to capture your data.

Thinking about the last time you used The Mix helpline, please tell us how strongly you agree or disagree with the following statements. *

Strongly disagree Tend to disagree Tend to agree Strongly agree Don't know Not applicable

I felt listened to.

I felt understood.

I felt emotionally supported.

What could we have done to support you even better?

How many times did you attempt to contact the helpline before you succeeded in getting through? *

Only once

2

3

4

5 or more

Other (please specify):

Before you used the helpline were you clear about what to expect from the service? *

Yes

No

I don't know

Please tell us what was unclear

How strongly you agree or disagree with the following statements: As a result of contacting the helpline... *

Strongly disagree Tend to disagree Tend to agree Strongly agree Don't know Not applicable

...my well-being improved.

...I feel more able to cope with my situation or issue.

...I feel more capable to make decisions.

...I formed/ I will form a plan to make a positive change to my situation.

Were any organisations, services or information sources recommended to you by the helpline agent? *

Yes

No

I don't know

Did you find the below organisations or resources recommended to you useful?

Yes No I haven't used it but I'M planning to do so I haven't used it and NOT planning to do so Not relevant

[variable(sp1name)]

Did you find the below organisations or resources recommended to you useful?

Yes No I haven't used it but I'M planning to do so I haven't used it and NOT planning to do so Not relevant

[variable(sp1name)]

[variable(sp2name)]

Did you find the below organisations or resources recommended to you useful?

Yes No I haven't used it but I'M planning to do so I haven't used it and NOT planning to do so Not relevant

[variable(sp1name)]

[variable(sp2name)]

[variable(sp3name)]

How would you rate the helpline service overall? *

Excellent Good Satisfactory Poor Very poor

Did the service help you? *

Yes

No

Please tell us how the service helped you.

How can we improve?

Please let us know if you have any other comments or feedback.

We’d like to use anonymous quotes from this survey on The Mix website or in reports to enable other people to better understand the service. Please tick the option below if we can use anonymous quotes from this survey.... (Please select all that apply). *

On The Mix website

In reports

None of the above

Can we contact you to send you the prize if you won? *

Yes

No

Appendix S2

**Codebook for Qualitative Analysis**

**Theme 1: Feedback on the Choice of Signposts**

Definition

Comments specific to the signposts that influence young people’s perception on whether signposting was effective.

Sub-Themes

- **Relevance** - Whether the signposts were relevant to the young people’s specific concerns
- **Personalised** - Whether the signposts were specific to the young people’s demographics (e.g., geographical location, age, support needs)
- **Expectation** - Whether the signposts met young people’s other expectations (e.g., number, features, specific requests)

What Theme 1 is NOT

- Perception on the effectiveness of signposting (Coded to Theme 2)
- Reported effectiveness of signposts beyond the chats (Coded to Theme 4)

**Theme 2: Feedback on the Signposting Experience**

Definition

Comments specific to how signposting was completed by helpline volunteers.

Examples

- **Integration of Emotional Support with Signposting** – How signposting was integrated with offering emotional support by the volunteers
- **Elaboration on Signposts** – Whether sufficient details of signposts were provided to young people
- **Preference for Additional Support** – Preference for receiving additional support beyond signposting, such as making direct referrals internally or externally

What Theme 2 is NOT

- Whether the signposts themselves were effective (Coded to Theme 1)
- How signposting elicited emotional responses in young people (Coded to Theme 3)
- How signposting improved young people's understanding of resources (Coded to Theme 4)

**Theme 3: Responses to Signposting**

Definition

Young people’s immediate responses to signposting during or shortly after the chat.

Sub-Themes

- **Hopefulness and Direction** - Signposting inspiring hope and a sense of direction
- **Rejection and Overwhelm** – Signposting bringing feelings of rejection or overwhelm

What Theme 3 is NOT

- Opinions on whether the signposts were appropriate (Coded to Theme 1)
- Opinions on how signposting should be completed (Coded to Theme 2)
- How the signposts have/have not benefited young people after they engaged with the signposts (Coded to Theme 4)

**Theme 4: Effectiveness of Signposting**

Definition

Effects of signposting on young people's wellbeing after the chat has ended.

Sub-Themes

- **Insights into Resources** - Enhanced knowledge of resources for young people
- **Empowerment** – Enabling young people to learn more about their situation and seek support
- **Access to Signposts** – Ease for young people to access the signposted resources
- **Impacts on Wellbeing** – Long-term mental health outcomes from engaging with signposts

What Theme 4 is NOT

- Whether helpline volunteers have chosen the relevant signposts (Coded to Theme 1)
- Temporary boost in wellbeing shortly after being signposted resources (Coded to Theme 3)

## Appendix S3

**Individual Multinomial Logistic Regressions on Signposting Outcomes and Mode of Delivery, Gender, Ethnicity or Age**

**Table B1**

*Multinomial Logistic Regression on Signposting Outcomes and Mode of Delivery (n = 822)*

| **Predictors** | **Useful: No** | | | **Useful: Not Relevant** | | | **Useful: Not used and no intent** | | | **Useful: Not used but has intent** | | |
| --- | --- | --- | --- | --- | --- | --- | --- | --- | --- | --- | --- | --- |
|  | Odds Ratios | 95% CI | *P* | Odds Ratios | 95% CI | *P* | Odds Ratios | 95% CI | *P* | Odds Ratios | 95% CI | *P* |
| Intercept (Webchat) | 0.36 | 0.25 – 0.52 | **<.001** | 0.25 | 0.16 – 0.38 | **<.001** | 0.50 | 0.36 – 0.69 | **<.001** | 1.25 | 0.97 – 1.61 | .084 |
| Contact Form vs. Webchat | 1.24 | 0.67 – 2.32 | .493 | 3.02 | 1.66 – 5.50 | **<.001** | 2.16 | 1.30 – 3.59 | **.003** | 1.78 | 1.17 – 2.71 | **.007** |
| Email vs. Webchat | 1.93 | 1.01 – 3.68 | **.046** | 0.36 | 0.10 – 1.28 | .114 | 2.12 | 1.19 – 3.78 | **.011** | 1.19 | 0.71 – 1.98 | .507 |
| Phone vs. Webchat | 3.00 | 1.26 – 7.13 | **.013** | 0.33 | 0.04 – 2.68 | .301 | 0.83 | 0.28 – 2.49 | .744 | 1.00 | 0.45 – 2.23 | 1.000 |

*Note.* CI = confidence interval. Significant chi-square difference test (*F*(12) = 51.21, *P* < .001) indicated that the model was likely a good fit to the data. Nagelkerke's R^2^ = 0.288. AIC = 2412. BIC = 2487.

**Table B2**

*Multinomial Logistic Regression on Signposting Outcomes and Gender (n = 684)*

| **Predictors** | **Useful: No** | | | **Useful: Not used and no intent** | | | **Useful: Not used but has intent** | | | **Useful: Not Relevant** | | |
| --- | --- | --- | --- | --- | --- | --- | --- | --- | --- | --- | --- | --- |
|  | Odds Ratios | 95% CI | *P* | Odds Ratios | 95% CI | *P* | Odds Ratios | 95% CI | *P* | Odds Ratios | 95% CI | *P* |
| Intercept (Female) | 0.59 | 0.44 – 0.79 | **<.001** | 0.24 | 0.16 – 0.36 | **<.001** | 0.64 | 0.48 – 0.85 | **.002** | 1.42 | 1.13 – 1.78 | **.003** |
| Male vs. Female | 0.58 | 0.28 – 1.22 | .152 | 1.69 | 0.79 – 3.61 | .173 | 1.17 | 0.64 – 2.13 | .604 | 0.95 | 0.57 – 1.58 | .841 |
| Non-Binary/Transgender vs. Female | 0.00 | 0.00 – Inf | .992 | 0.00 | 0.00 – Inf | .992 | 0.45 | 0.09 – 2.20 | .322 | 0.50 | 0.16 – 1.63 | .252 |
| Other vs. Female | 0.00 | 0.00 – Inf | .990 | 0.00 | 0.00 – Inf | .991 | 4.69 | 1.23 – 17.84 | **.023** | 3.06 | 0.85 – 10.96 | .086 |
| Prefer not to say vs. Female | 0.00 | 0.00 – Inf | .987 | 5.36 | 1.85 – 15.54 | **.002** | 1.56 | 0.53 – 4.62 | .420 | 1.31 | 0.51 – 3.38 | .575 |

*Note.* CI = confidence interval. Significant chi-square difference test (*F*(16) = 51.67, *P* < .001) indicated that the model was likely a good fit to the data. Nagelkerke's R^2^ = 0.298. AIC = 2011. BIC = 2102. Non-binary (*n* = 8) and transgender (*n* = 6) were collapsed due to small sample sizes.

**Table B3**

*Multinomial Logistic Regression on Signposting Outcomes and Ethnicity (n = 669)*

| **Predictors** | **Useful: No** | | | **Useful: Not used and no intent** | | | **Useful: Not used but has intent** | | | **Useful: Not Relevant** | | |
| --- | --- | --- | --- | --- | --- | --- | --- | --- | --- | --- | --- | --- |
|  | Odds Ratios | 95% CI | *P* | Odds Ratios | 95% CI | *P* | Odds Ratios | 95% CI | *P* | Odds Ratios | 95% CI | *P* |
| Intercept (White) | 0.42 | 0.31 – 0.57 | **<.001** | 0.29 | 0.21 – 0.41 | **<.001** | 0.70 | 0.54 – 0.90 | **.006** | 1.23 | 0.99 – 1.53 | .065 |
| Asian vs. White | 2.38 | 0.94 – 6.02 | .066 | 1.36 | 0.41 – 4.56 | .617 | 1.57 | 0.64 – 3.84 | .320 | 2.52 | 1.19 – 5.31 | **.015** |
| Black vs. White | 1.19 | 0.29 – 4.92 | .809 | 0.57 | 0.07 – 4.85 | .605 | 1.43 | 0.45 – 4.56 | .546 | 1.22 | 0.42 – 3.50 | .714 |
| Mixed vs. White | 1.36 | 0.38 – 4.82 | .632 | 1.46 | 0.36 – 5.89 | .596 | 0.82 | 0.23 – 2.87 | .752 | 2.09 | 0.85 – 5.14 | .109 |
| Other vs. White | 2.38 | 0.00 – Inf | 1.000 | 3.40 | 0.00 - Inf | 1.000 | 1.43 | 0.00 – Inf | 1.000 | 13505156.27 | 0.00 – Inf | .994 |
| Prefer not to say vs. White | 16.68 | 2.01 – 138.56 | **.009** | 0.00 | 0.00 - Inf | .988 | 1.43 | 0.09 – 23.13 | .801 | 7.31 | 0.92 – 58.40 | .061 |

*Note.* CI = confidence interval. Significant chi-square difference test (*F*(20) = 65.79, *P* = .020) indicated that the model was likely a good fit to the data. Nagelkerke's R^2^ = 0.280. AIC = 2000. BIC = 2100.

**Table B4**

*Multinomial Logistic Regression on Signposting Outcomes and Age (n = 697)*

| **Predictors** | **Useful: No** | | | **Useful: Not used and no intent** | | | **Useful: Not used but has intent** | | | **Useful: Not Relevant** | | |
| --- | --- | --- | --- | --- | --- | --- | --- | --- | --- | --- | --- | --- |
|  | Odds Ratios | 95% CI | *P* | Odds Ratios | 95% CI | *P* | Odds Ratios | 95% CI | *P* | Odds Ratios | 95% CI | *P* |
| Intercept (16-19) | 0.39 | 0.27 – 0.56 | **<.001** | 0.21 | 0.14 – 0.33 | **<.001** | 0.56 | 0.41 – 0.77 | **<.001** | 1.24 | 0.96 – 1.60 | .095 |
| 20-21 vs. 16-19 | 1.81 | 0.91 – 3.60 | .090 | 2.26 | 1.02 – 5.03 | **.046** | 2.43 | 1.35 – 4.36 | **.003** | 1.61 | 0.95 – 2.73 | .076 |
| 22-25 vs. 16-19 | 1.82 | 0.95 – 3.50 | .071 | 2.42 | 1.14 – 5.14 | **.021** | 1.26 | 0.67 – 2.36 | .478 | 1.59 | 0.96 – 2.62 | .071 |
| 26+ vs. 16-19 | 0.57 | 0.12 – 2.76 | .486 | 0.52 | 0.06 – 4.32 | .546 | 0.79 | 0.23 – 2.66 | .700 | 0.63 | 0.23 – 1.74 | .369 |
| Prefer not to say vs. 16-19 | 0.00 | 0.00 – Inf | .985 | 0.00 | 0.00 – Inf | .986 | 0.00 | 0.00 – Inf | .985 | 0.27 | 0.03 – 2.62 | .258 |

*Note.* CI = confidence interval. Marginally significant chi-square difference test (*F*(16) = 24.30, *P* = .083) indicated that the model was likely a good fit to the data. Nagelkerke's R^2^ = 0.271. AIC = 2073. BIC = 2164.
