# Supplementary material for: Young People’s Perceptions of Signposting in a Digital Mental Health Helpline: Mixed Methods Analysis of Cross-Sectional Data
Source: JMIR Hum Factors. 2026 May 19;13:e73369. doi: 10.2196/73369 (PMC13186521; doi:10.2196/73369)
Supplement: Checklist 1 [file humanfactors-v13-e73369-s002.pdf]

CHERRIES Checklist

CHERRIES Item	Reported in This Manuscript	Location in Manuscript
Design (open vs closed; context)	Closed, post-contact web survey of The Mix service users across webchat, email, telephone, and web contact form; service context described.	Methods – Setting / Participants
Survey timeframe	February 2020 to October 2023.	Methods – Setting
Recruitment process & contact mode	Email invitations sent to recent helpline users with a survey link; participation voluntary.	Methods – Participants
Incentives	Entry into a £50 voucher raffle.	Methods – Participants
Ethics approval (IRB/REC)	UCL Research Ethics Committee approval (ID: 16583/003).	Methods – Ethics
Informed consent	Online information sheet; informed consent obtained before any items.	Methods – Procedure / Ethics
Data protection / privacy	Not reported.	—
Questionnaire development / pretesting	Not reported.	—
Survey platform / software	Not reported.	—
Randomization of items/questionnaires	Not reported.	—
Adaptive questioning / branching (skip logic)	Not reported.	—
Number of items (questions)	Not reported.	—
Number of pages/screens	Not reported.	—
Completeness checks (forced responses/prompts)	Not reported.	—
Review step (ability to change answers)	Not reported.	—
Preventing multiple entries (cookies/IP/logs/unique links)	Not reported beyond closed invitation; no specific technical method described.	Methods – Participants (note)
View rate (unique site visitors / survey visitors)	Not applicable (closed survey).	Methods – Data Analysis (CHERRIES note)

CHERRIES Item	Reported in This Manuscript	Location in Manuscript
Participation rate (consented / invited)	Not reported numerically.	Methods – Data Analysis (CHERRIES note)
Completion rate (completed / consented)	Not reported numerically.	Methods – Data Analysis (CHERRIES note)
Handling of incomplete questionnaires / missing data	Observations with missing demographic data removed within each predictor analysis; partial outcomes excluded where applicable (analytical samples as stated).	Methods – Data Analysis / Results
Statistical correction (e.g., weighting)	Not applicable / Not reported.	—
Primary analyses	Multinomial logistic regression (reference category: “Used and found useful”).	Methods – Quantitative Analysis
Qualitative analyses	Inductive thematic analysis of open-ended responses.	Methods – Qualitative Analysis
